# Upregulation of Nucleotide-Binding Oligomerization Domain-, LRR- and Pyrin Domain-Containing Protein 3 in Motoneurons Following Peripheral Nerve Injury in Mice

**DOI:** 10.3389/fphar.2020.584184

**Published:** 2020-11-26

**Authors:** Bernát Nógrádi, Ádám Nyúl-Tóth, Mihály Kozma, Kinga Molnár, Roland Patai, László Siklós, Imola Wilhelm, István A. Krizbai

**Affiliations:** ^1^ Institute of Biophysics, Biological Research Centre, Szeged, Hungary; ^2^ Foundation for the Future of Biomedical Sciences in Szeged, Szeged Scientists Academy, Szeged, Hungary; ^3^ Department of Biochemistry and Molecular Biology, Vascular Cognitive Impairment and Neurodegeneration Program, Reynolds Oklahoma Center on Aging/Oklahoma Center for Geroscience, University of Oklahoma Health Sciences Center, Oklahoma City, OK, United States; ^4^ Theoretical Medicine Doctoral School, University of Szeged, Szeged, Hungary; ^5^ Institute of Life Sciences, Vasile Goldiş Western University of Arad, Arad, Romania

**Keywords:** acute nerve injury, motor neuron, neuroinflammation, inflammasome, nucleotide-binding oligomerization domain-, LRR- and pyrin domain-containing protein 3, diazoxide

## Abstract

Neuronal injuries are accompanied by release and accumulation of damage-associated molecules, which in turn may contribute to activation of the immune system. Since a wide range of danger signals (including endogenous ones) are detected by the nucleotide-binding oligomerization domain-, LRR- and pyrin domain-containing protein 3 (NLRP3) pattern recognition receptor, we hypothesized that NLRP3 may become activated in response to motor neuron injury. Here we show that peripheral injury of the oculomotor and the hypoglossal nerves results in upregulation of NLRP3 in corresponding motor nuclei in the brainstem of mice. Although basal expression of NLRP3 was observed in microglia, astroglia and neurons as well, its upregulation and co-localization with apoptosis-associated speck-like protein containing a caspase activation and recruitment domain, suggesting inflammasome activation, was only detected in neurons. Consequently, increased production of active pro-inflammatory cytokines interleukin-1β and interleukin-18 were detected after hypoglossal nerve axotomy. Injury-sensitive hypoglossal neurons responded with a more pronounced NLRP3 upregulation than injury-resistant motor neurons of the oculomotor nucleus. We further demonstrated that the mitochondrial protector diazoxide was able to reduce NLRP3 upregulation in a post-operative treatment paradigm. Our results indicate that NLRP3 is activated in motoneurons following acute nerve injury. Blockade of NLRP3 activation might contribute to the previously observed anti-inflammatory and neuroprotective effects of diazoxide.

## Introduction

Finely tuned interaction between the nervous and the immune systems and different inflammatory processes play a pivotal role in the consequences of neuronal injury. The innate immune system-mediated inflammation can act both as detrimental (reviewed in Labzin et al., 2018; Schwartz et al., 2016) or beneficial player after insult to the central nervous system (CNS) (Raposo et al., 2014), thus may influence the fate of affected neurons after the lesion. Sensing of potentially dangerous molecular structures by the innate immune system relies on pattern recognition receptors (PRRs), whose activation can lead to the induction of inflammatory processes. PRRs are mainly expressed in immune cells; however, cellular components of the CNS, including neurons, have also been demonstrated to express PRRs.

Ligand recognition by several members of the nucleotide-binding oligomerization domain (NOD)-like receptor (NLR) family leads to activation of a multiprotein complex, the inflammasome, which recruits and activates caspase-1 through the adaptor molecule apoptosis-associated speck-like protein containing a CARD (ASC) to proteolytically maturate interleukin-1 beta (IL-1β) and IL-18 (IL-18). The most important NLRs in this respect are NOD, leucine rich repeat and pyrin domain containing (NLRP) 1, nucleotide-binding oligomerization domain NOD-, LRR- and pyrin domain-containing protein 3 (NLRP3), and NLR family caspase activation and recruitment domain (CARD)-containing domain (NLRC) 4. In addition, absent in melanoma 2 (AIM2) can also be part of inflammasomes (Latz et al., 2013). Despite an acknowledged role of inflammation in a large number of CNS disorders, only a few inflammasomes have been characterized so far in the CNS. These include NLRP3, NLRP1 and NLRC4 inflammasomes in microglia and astrocytes (Abulafia et al., 2008; Halle et al., 2008; Hanamsagar et al., 2011; Liu et al., 2013; Freeman et al., 2017) and NLRP2 inflammasome in astrocytes (Minkiewicz et al., 2013). In neurons, initially NLRP1 (Abulafia et al., 2008; de Rivero Vaccari et al., 2008) and AIM2 (Adamczak et al., 2014) inflammasomes were described to be activated in response to different stimuli. In addition, neurons can also express NLRP3 (von Herrmann et al., 2018) to initiate inflammasome formation (Panicker et al., 2020), which is by far the most investigated inflammasome in the CNS. Nowadays it is clear that NLRP3 plays an active role in the pathomechanism of a wide variety of neurological diseases and aging (Latz et al., 2018; Mészáros et al., 2020; Heneka et al., 2013), especially in microglia. Much less is known about regulation of NLRP3 in neurons in response to acute injury.

In humans, peripheral nerve injuries usually occur in traumatic accidents and often lead to complications, such as chronic pain and motor or sensory loss of function (Althagafi and Nadi 2020). In mice, axotomy is a well-documented and standardized method to induce motoneuronal injury (e.g., Koliatsos and Price 1996). Axonal lesions lead to retrograde changes in neurons, activation of glial cells and inflammatory reactions, including extensive microgliosis (Rotterman and Alvarez 2020). Our goal was to determine whether NLRP3-mediated processes could occur in response to peripheral nerve axotomy. Oculomotor and hypoglossal neurons were selected for our experiments because of the difference in the neuronal vulnerability and in the inflammatory response to injury (Obál et al., 2006; Nógrádi et al., 2020).

Mitochondrial dysfunction is a key component and regulator of NLRP3-mediated inflammasome activation (Liu et al., 2018). Furthermore, activated NLRP3 may translocate to mitochondria-associated endoplasmic reticulum membranes, which provide a platform for NLRP3 inflammasome assembly. On the other hand, NLRP3 activators induce mitochondrial damage, while NLRP3 directly interacts with molecules released from injured mitochondria, like cardiolipin, mitochondrial DNA and reactive oxygen species (Elliott et al., 2018; Zhong et al., 2018). Therefore, possible effect of diazoxide (7-chloro-3-methyl-4H-1λ6,2,4-benzothiadiazine 1,1-dioxide), a mitochondrial K^+^_ATP_ channel opener and neuroprotective agent, on inflammasome activation was assessed as well.

## Material and Methods

### Ethical Considerations

All efforts were made to minimize animal suffering throughout the experiments, thus multiple surgeries were avoided. All experiments were carried out in accordance with the institutional guidelines for the use and care of the experimental animals and governmental law for animal protection. The experimental protocols and the animal care were approved by the institutional care and the Regional Animal Health and Food Control Station of Csongrád-Csanád County (permit number: XVI./767/2018 and 03876/0014/2006) and carried out in accordance with the national law (XXVIII. chapter IV. paragraph 31) which conforms to the international laws and policies (EEC Council Directive 86/609, OJL 358 1 DEC. 12, 1987; NIH Guide for the Care and Use of Laboratory Animals, United States National Research Council, revised 1996).

### Experimental Animals and Surgical Procedures

Balb/c non-transgenic mice (mean body weight of 22 ± 5 g) were housed in the conventional animal facility of the Biological Research Center (Szeged, Hungary) for the period of the experiments. Examinations were performed on 33 young adult (8–15 week old) male mice, housed in plastic cages (three animals per cage at most) in a thermoneutral (23 ± 2°C) room under a 12 h light:dark cycle, with access to regular rodent chow and water *ad libitum*.

To avoid multiple surgical procedures on individual animals, mice were assigned either to eye enucleation (target deprivation) or hypoglossal nerve axotomy. A total of 30 mice were used for immunohistochemistry (IHC) quantification. From these animals, 10 served as non-operated controls (n = 5 for the oculomotor nucleus, n = 5 for the hypoglossal nucleus) and 20 animals underwent either eye enucleation [n = 5 (non-treated)] or hypoglossal nerve axotomy [n = 15 (n = 5 non-treated, n = 5 diazoxide-treated, n = 5 dimethyl sulfoxide/(DMSO)-treated)]. For western blot (WB) evaluation of IL-1β and IL-18 pro-inflammatory cytokines, surgical unilateral axotomy of the hypoglossal nerve was conducted on three animals. Surgical interventions were performed under deep and reversible anesthesia with Avertin (tribromoethanol, Sigma-Aldrich; 240 mg/kg body weight in a 0.5 ml volume), administered intraperitoneally. For target deprivation of the oculomotor nerve, animals were enucleated, the right eyeball, remaining extraocular muscles and lacrimal gland were removed carefully from the orbit. In case of hypoglossal axotomy, 1 cm long midline incision was made below the hyoid bone, the right cranial nerve XII. was carefully prepared and a 2–3 mm nerve segment was dissected to prevent regeneration.

For diazoxide treatment, 0.25 mg/ml diazoxide (Sigma-Aldrich) was dissolved in 10 mg/ml DMSO (Sigma-Aldrich) in distilled water in a 0.1 ml volume and was administered by intraperitoneal injection (1 mg/kg bodyweight). DMSO-treatment was used as vehicle control (10 mg/ml DMSO dissolved in distilled water, administered intraperitoneally). Animals received the first dose 3 h after the axotomy, then on the first, second and third postoperative days (every 24 h, altogether four doses). On the fourth postoperative day, animals were sacrificed, thus received no treatment. Axotomized, non-treated mice received no treatment. All animals were allowed to survive for 4 days. In each case, the non-operated side of the motor nucleus served as an internal control to determine the difference in NLRP3 (IHC) or IL-1β (WB) expression.

### Immunohistochemistry and Immunofluorescence Staining Procedures

Under irreversible anesthesia with Avertin, mice were transcardially perfused with 10 mM phosphate buffered saline (PBS; pH 7.4) followed by 4% paraformaldehyde (Sigma-Aldrich) in 10 mM PBS (pH 7.4). The entire brain was exposed and removed, then fixed further overnight in the same fixative at 4°C. After the fixation protocol, samples were cryoprotected in 30% sucrose (Sigma-Aldrich) dissolved in 10 mM PBS, for at least 1 day at 4°C. Series of consecutive coronal sections of 30 µm thickness were cut throughout the whole anatomical regions of interests with a microtome (Reichert-Jung, Leica Biosystems), collected in 10 mM PBS individually in wells of tissue culture plates and stored at 4°C until processed for staining.

IHC stainings were performed on 30 µm thick free-floating sections. For the quantitative assessment of the changes in NLRP3, diaminobenzidine tetrahydrochloride (DAB)-based IHC was used. In both examined motor nuclei, sections (n = 8/each animal) were selected with respect to the anatomical boundaries. Sections were rinsed (three changes, 5 min each), then 50% methanol (VWR Chemicals) in 10 mM PBS was applied for tissue permeabilization for 30 min at −20°C. Samples were rinsed again in 10 mM PBS, then the non-specific staining was blocked in two steps: 0.6% hydrogen peroxide in 10 mM PBS containing 0.2% Triton X-100 (Sigma-Aldrich) (TPBS) was used first for 30 min to block the endogenous peroxidase activity. After sections were rinsed (three changes, 5 min), the second blocking step was applied with 2% normal rabbit serum (Vector Laboratories) in 10 mM TPBS for 1 h. This was followed by overnight incubation at 4°C with the polyclonal goat primary antibody against NLRP3 diluted in 10 mM TPBS with 2% normal rabbit serum. After washing in 10 mM PBS (three changes, 5 min), sections were incubated at room temperature in a biotinylated rabbit-anti-goat secondary antibody diluted in 10 mM TPBS with 2% normal rabbit serum for 1 h. Next, all the sections were rinsed in 10 mM PBS (three changes, 5 min each), incubated in avidin-biotin complex (Vector Laboratories) diluted to 1:800 in PBS for 1 h at room temperature. After washing in 10 mM PBS, the reactions were visualized by incubation in 0.5% DAB (Sigma-Aldrich) with 1.5% NiCl_2_ in 10 mM PBS for 15 min. Finally, sections were washed in 10 mM PBS (three changes, 5 min each), mounted on silane-coated glass slides, covered with Entellan (Merck Millipore) and visualized under a brightfield microscope (Eclipse 80i, Nikon). Brightness and contrast were adjusted as needed. To qualitatively evaluate changes in the level of the inflammasome component ASC, the IHC procedure was carried out as previously described, with the difference that serum-specific blocking was performed with 2% normal goat serum (Jackson Immunoresearch).

Immunofluorescence (IF) staining protocols were also performed on 30 µm thick free-floating sections. First, selected sections were rinsed, then 50% methanol was applied, as previously described. Sections were rinsed again (three changes, 5 min each), then a blocking step was used with 2% normal donkey serum (Jackson Immunoresearch) in 10 mM TPBS for 1 h. Primary antibody cocktails were applied for overnight incubation at 4°C. The next day, sections were rinsed (three changes, 5 min each) and secondary antibody cocktails were used for 1 h. Both the primary and secondary cocktails were diluted in 10 mM TPBS with 2% normal donkey serum. Primary and secondary antibody cocktails varied in each double or triple immunofluorescent staining procedure and are detailed in Table 1. Finally, sections were washed in 10 mM PBS (three changes, 5 min each) and, where indicated, Hoechst 33342 (B2261, Sigma-Aldrich) staining was applied (diluted to 1 μg/ml in 10 mM PBS) to visualize cell nuclei. Sections were mounted on silane-coated glass slides, covered with Fluoromount-G (0100-01, Southern Biotechnology Associates) mounting medium. Secondary antibody staining controls have been carried out for each of the applied secondary antibodies to exclude the interference of any associated unspecific staining.

**TABLE 1:** Antibodies used for IHC, IF and WB.

Stainings	Primary antibodies	Secondary antibodies
NLRP3 (IHC)	Polyclonal goat against **NLRP3**, 1:500 (GTX88190, GeneTex)	Biotinylated rabbit anti-goat IgG antibody (H + L), 1:800 (BA-5000, Vector Laboratories)
ASC (IHC)	Monoclonal mouse against **ASC**, 1:400 (sc-271054, Santa Cruz Biotechnology)	Biotinylated goat anti-mouse IgG antibody (H + L), 1:800 (BA-5000, Vector Laboratories)
NLRP3, NeuN (IF) and hoechst	Anti-**NLRP3**, 1:400	Alexa Fluor^®^ 488 cross-adsorbed donkey anti-goat IgG, 1:500 (A-11055, Thermo Fisher Scientific)
	Polyclonal mouse against **NeuN**, 1:500 (MAB377, Millipore)	Cy™5 AffiniPure donkey anti-mouse IgG (H + L), 1:500 (715–175-150, Jackson Immunoresearch)
NLRP3, ChAT (IF) and hoechst	Anti-**NLRP3**, 1:400	Cy™3 AffiniPure donkey anti-goat IgG (H + L), 1:500 (705–165-003, Jackson Immunoresearch)
	Polyclonal rabbit against **ChAT**, 1:250 (GTX113164, GeneTex)	Alexa Fluor^®^ 488 AffiniPure donkey anti-rabbit IgG (H + L), 1:500 (711–545-152, Jackson Immunoresearch)
NLRP3, GFAP (IF) and hoechst	Anti-**NLRP3**, 1:400	Alexa Fluor^®^ 488 donkey anti-goat IgG (H + L), 1:500
	Polyclonal rabbit against **GFAP**, 1:500 (ab7260, Abcam)	Alexa Fluor^®^ 546 highly cross-adsorbed donkey anti-rabbit IgG (H + L), 1:500 (A-10040, Thermo Fisher Scientific)
NLRP3, IBA1 (IF) and hoechst	Polyclonal goat against **NLRP3**, 1:400 (GTX88190, GeneTex)	Alexa Fluor^®^ 488 donkey anti-goat IgG (H + L), 1:500
	Polyclonal rabbit against **IBA1**, 1:500 (019–19,741, Wako)	Alexa Fluor^®^ 546 donkey anti-rabbit IgG, 1:500
NLRP3 and AQP4 (IF)	anti-**NLRP3**, 1:400	Cy™3 donkey anti-goat IgG (H + L), 1:500
	Polyclonal rabbit against **AQP4**, 1:100, (sc-390488, Santa Cruz Biotechnology)	Alexa Fluor^®^ 647 AffiniPure donkey anti-rabbit IgG (H + L), 1:500 (711-605-152, Jackson Immunoresearch)
NLRP3, GFAP and ASC (IF)	Anti-**NLRP3**, 1:400	Alexa Fluor^®^ 488 cross-adsorbed donkey anti-goat IgG, 1:500 (A-11055, Thermo Fisher Scientific)
	Anti-**GFAP**, 1:500	Alexa Fluor^®^ 546 donkey anti-rabbit IgG, 1:500
	Anti-**ASC**, 1:100	Cy™5 donkey anti-mouse IgG, 1:500
IL-1β (WB)	Polyclonal goat against **IL-1β**, 1:500 (AF-401-NA, R&D Systems)	HRP-conjugated rabbit anti-goat IgG (H + L), 1:4,000 (A5420, Sigma-Aldrich)
IL-18 (WB)	Polyclonal rabbit against **IL-18**, 1:500 (5180R-100, BioVision Incorporated)	HRP-conjugated goat anti-rabbit IgG (H + L), 1:4,000 (111–035-003, Jackson Immunoresearch)
β-actin (WB)	Monoclonal mouse against **β-actin**, 1:10,000 (A5441, Sigma-Aldrich)	HRP-conjugated goat anti-mouse IgG (H + L), 1:4,000 (115–035-003, Jackson Immunoresearch)

NLRP3, nucleotide-binding oligomerization domain-, LRR- and pyrin domain-containing protein 3; ASC, apoptosis-associated speck-like protein containing a caspase activation and recruitment domain; IF, immunofluorescence; IHC, immunohistochemistry; WB, western blot; IL-1β; interleukin-1 beta; IL-18, interleukin-18; GFAP, glial fibrillary acidic protein.

### Confocal and Super-Resolution Microscopy

Immunofluorescence co-staining was examined with confocal and super-resolution microscopy (STED) (stimulated emission depletion) microscopy. Lower resolution imaging was performed on a Leica SP5 Laser Scanning Microscope (Leica Microsystems), while super-resolution images were obtained with a STEDYCON (Abberior Instruments) STED instrument connected to a ZEISS Axio Observer Z1 inverted microscope. From raw images, confocal z-stacks were prepared with LAS X viewer and FIJI (ImageJ 1.51n engine) software. Some color images are presented in false color, in order to simplify visualization of certain stainings by presenting the same target proteins in uniform colors throughout different images. Brightness and contrast were adjusted as needed.

### Quantitative Evaluation of Light Microscopy Immunohistochemistry Staining

Quantitative assessment of NLRP3 expression was carried out with the use of the DAB-based IHC technique, as this method provides photostability, in contrast to IF. Selection of sections for staining and evaluation was performed following a systematic regime. Anatomical boundaries of the nuclei were considered and eight sections were stained for quantification in each nucleus, in a way that after a section was selected, the consecutive section was excluded, to cover more of the anatomical region along the rostrocaudal axis of the brainstem. The contralateral (control) side of the samples was marked with a small incision during the sectioning, thus the injured and control sides could be clearly distinguished during the evaluation. After staining the series of the sections, a standardized digital image recording (in a Nikon Eclipse 80i microscope equipped with a 2,560 × 1,920 pixel resolution MicroPublisher 5.0 RTV charge-coupled device camera, QImaging) was conducted on all sections at ×10 magnification, thus both the operated and contralateral (control) sides of the nucleus were recorded on the same image. As the first step of the image analysis protocol, a consistent background subtraction algorithm was applied, based on internal controls (contralateral side) in each section, to determine the significantly stained profiles in identical regions at both the operated and contralateral sides of the nuclei. This was performed by using an interactive macro developed in our laboratory (Nógrádi et al., 2020) for the Image-Pro Plus image analysis software (Media Cybernetics).This resulted in an automated, unbiased evaluation procedure, since the algorithm determined the stained profile values with the same background subtraction for both the injured and contralateral sides on each section. As the significantly stained partial profile areas were determined at both sides of the brainstem, the data were expressed as a percentage, and the algebraic differences between the operated and the contralateral sides were determined. These numbers were averaged for the stained sections, to arrive at a single number characterizing the net change in the area covered by the significant immunoreaction induced by the axotomy in each animal.

To quantify the ratio of neurons in which the translocation of the target protein from the cell nuclei to the cytoplasm could be observed, a cell-counting procedure was carried out. From the non-treated axotomized group (hypoglossal axotomy), sections were selected from the hypoglossal nucleus of each animal (n = 5 animals). Neuronal cells were recognized based on their size and the anatomical boundaries. In each of the sections, NLRP3 positive neurons were counted based on the localization of the staining. If NLRP3 was only present in the neuronal nucleus, the expression was counted as “nuclear NLRP3.” If NLRP3 staining was clearly present in the cytoplasm, the expression was counted as “cytoplasmic NLRP3.” When NLRP3 was clearly present in both the cytoplasm and nucleus, cells were sorted in the “cytoplasmic NLRP3” group, since the nuclear NLRP3 expression was recognized as the basal expression and the cytoplasmic presence of NLRP3 indicated translocation. Neurons were only counted if a well-described point of reference (cell nucleus) could be recognized in the section. Neurons were counted on both the operated and the control side. The ratio of translocation was determined for each side of each section and was averaged.

### Sample Preparation, Methanol-Chloroform Precipitation and Western Blot

Under irreversible anesthesia with Avertin, mice were transcardially perfused with 10 mM PBS. The entire brain was exposed, removed and placed in 10 mM PBS, then dissected in the following manner: first, the brain was coronally sliced at approximately −6 mm from Bregma (at the *medulla oblongata* – *pons* transition), then at -7.5 mm from Bregma (at the appearance of the *decussatio pyramidum*). Next, the cerebellum was carefully dissected and removed from the sample. From the remaining sample, 1 mm wide lateral segments were sliced and removed along the sagittal plane on both sides. From the ventral part of the *medulla oblongata*, a 0.5 mm wide segment was cut and removed horizontally. Finally, the sample was sliced along the mid-sagittal axis and the two sides (injured and control sides) were separated and placed into sample holders.

Snap-freezing in liquid nitrogen was performed immediately after tissue dissection, then samples were processed in a Potter-Elvehjem homogenizer with a PTFE pestle in 10 mM PBS. Samples were grinded on ice until they were completely homogeneous. The homogenizer was thoroughly washed multiple times with distilled water between samples. Samples were centrifuged twice at 6,000 × g for 8 min at 4°C to remove debris. An equal volume of methanol and 1/4 volume of chloroform were added. Samples were vortexed, incubated for 5 min on ice, and centrifuged at 13,000 × g for 5 min at 4°C. After phase separation, aqueous phase was removed, and protein samples were washed with ice-cold methanol. Samples were vortexed and centrifuged again, supernatants were discarded, and protein pellets were air-dried. Pellets were reconstituted in 2× Laemmli buffer and heated up to 95°C for 5 min. Protein concentration was determined by using bicinchoninic acid assay (Thermo Fisher Scientific).

Samples were electrophoresed using standard denaturing SDS/PAGE and blotted on polyvinylidene difluoride membranes (0.2 μm pore size; Bio-Rad). After blocking with 3% bovine serum albumin (BSA) in Tris-buffered saline with 0.1% Tween-20 (TBS-T), membranes were incubated with primary antibodies (Table 1) overnight at 4°C. Blots were washed in TBS-T three times for 10 min, incubated for 1 h in horseradish peroxidase-conjugated secondary antibodies (Table 1) diluted in TBS-T, and then washed again in TBS-T. Immunoreaction was visualized with Clarity Chemiluminescence Substrate (Bio-Rad) in a ChemiDoc MP System (Bio-Rad). Densitometry analysis was performed with the Image lab software, version 5.2 (Bio-Rad).

### Statistical Analysis of the Data

Student’s t-test was applied to evaluate the differences in NLRP3 translocation from the nucleus to the cytoplasm and the WB quantification of the active IL-1β and IL-18 levels in the axotomized and control sides. Differences among the means of NLRP3 immunostaining was assessed by one-way ANOVA with Fisher LSD (least significant difference) post-hoc test. All statistical analysis was performed with R (version 3.6.2; R Foundation for Statistical Computing) and RStudio integrated development environment (RStudio). All data are represented as mean ± SEM. In order to determine the number of animals needed (sample size: n = 5 for IHC and n = 3 for WB), power analysis was carried out with G × Power (Faul et al., 2009).

## Results

### Expression of Nucleotide-Binding Oligomerization Domain-, LRR- and Pyrin Domain-Containing Protein 3 in Motor Neurons in Response to Target Deprivation in the Oculomotor Nucleus and Axotomy in the Hypoglossal Nucleus

First, we assessed NLRP3 expression in the brainstem of animals exposed to axotomy. According to literature data, in traumatic brain injury (TBI), the level of inflammasome proteins (NLRP3, ASC, caspase-1) starts to increase 6 h after injury and peaked at 3 and 7 days (Liu et al., 2013). Furthermore, our previous experiments demonstrated that microglial activation and morphological changes, which might correlate with the inflammatory peak (Fernández-Arjona et al., 2019), show highest intensity at 4 and 7 days following nerve axotomy (Paizs et al., 2017; Nógrádi et al., 2020). Thus, in our experiments, all animals were allowed to survive for 4 days until the peak immune/inflammatory reaction was observed.

**FIGURE 1:**
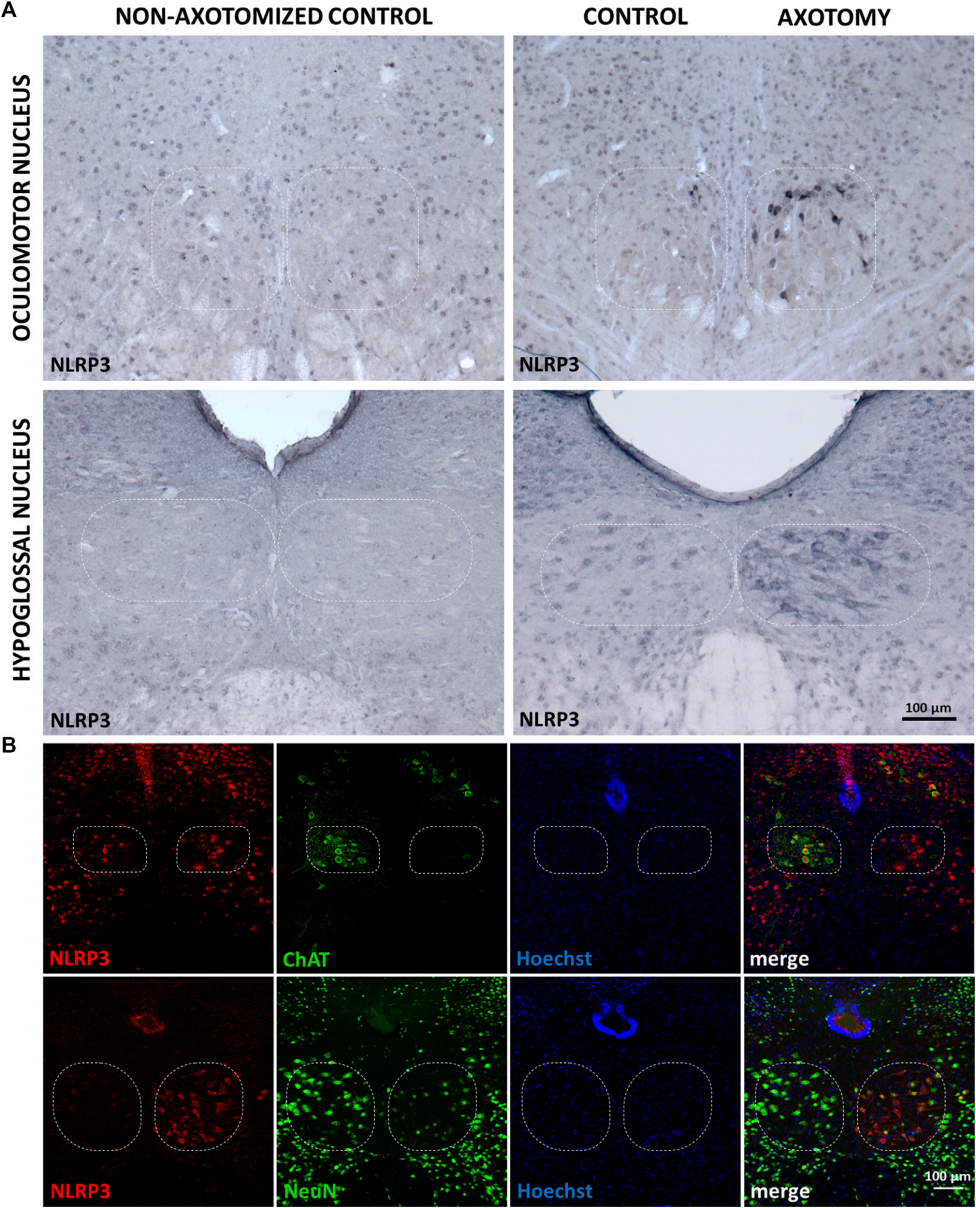
Nucleotide-binding oligomerization domain-, LRR- and pyrin domain-containing protein 3 (NLRP3) protein expression in motor neurons of the oculomotor and hypoglossal nuclei after specific nerve axotomy. **(A)** Representative immunohistochemistry stainings of NLRP3 protein on Balb/c mouse brain sections from the anatomical region of the oculomotor **(top panel)** and the hypoglossal **(bottom panel)** nuclei (scale: 100 µm). Corresponding brain nerves were axotomized. **(B)** Representative fluorescence immunostaining images of NLRP3, neuronal nuclei **(top panel)** and choline acetyltransferase **(bottom panel)** proteins on Balb/c mouse brain sections from the anatomical region of hypoglossal nucleus after corresponding brain nerve axotomy (scale: 100 µm). Nuclei were counterstained with Hoechst 33342.

Under control (i.e., non-operated) conditions, there was a faint basal NLRP3 staining in both sides of the oculomotor and hypoglossal nuclei in mouse brain sections. Target deprivation in the case of the oculomotor nucleus led to a significant increase in the NLRP3 staining which was absent in the contralateral side serving as an internal control (Figure 1A). Similar changes were visible in the hypoglossal nucleus after the transection of the hypoglossal nerve; however, the reaction was more intense (Figure 1A). Quantitative analysis revealed that increase in NLRP3 was significant in both the oculomotor nucleus (8.04 ± 1.98 vs. 2.66 ± 0.34%; injured *vs.* non-operated; *p* < 0.05) and the hypoglossal nucleus (27.25 ± 4.87 vs*.*1.13 ± 0.51%; *p* < 0.0005) after the nerve transection, when compared to the non-operated controls. Furthermore, NLRP3 increase was significantly lower in the oculomotor nucleus compared to the hypoglossal nucleus following axotomy (8.04 ± 1.98 vs*.* 27.25 ± 4.87%; injured oculomotor nucleus vs. injured hypoglossal nucleus; *p* < 0.0005).

Morphology of the staining suggested that the majority of the NLRP3-positive cells were neurons. Therefore, we performed co-stainings with neuronal nuclei (NeuN) and choline acetyltransferase (ChAT) markers (Figure 1B). We chose the hypoglossal nucleus because more intense reaction could be observed compared to the oculomotor nucleus, following axotomy. NeuN staining decreased in the affected nucleus compared to its contralateral counterpart reflecting a nucleus-specific neuronal response to target deprivation, as described by others (McPhail et al., 2004; Obál et al., 2006). As anticipated, a significant number of NLRP3-positive cells were NeuN positive as well (Figure 1B), indicating neuronal upregulation of NLRP3 in response to target deprivation. ChAT staining almost completely overlapped with NLRP3 staining, indicating that indeed motoneurons expressed NLRP3 (Figure 1B). However, at the side of the lesion, ChAT staining, similarly to NeuN staining, decreased due to the disturbance in the neuronal homeostasis (Lams et al., 1988).

**FIGURE 2:**
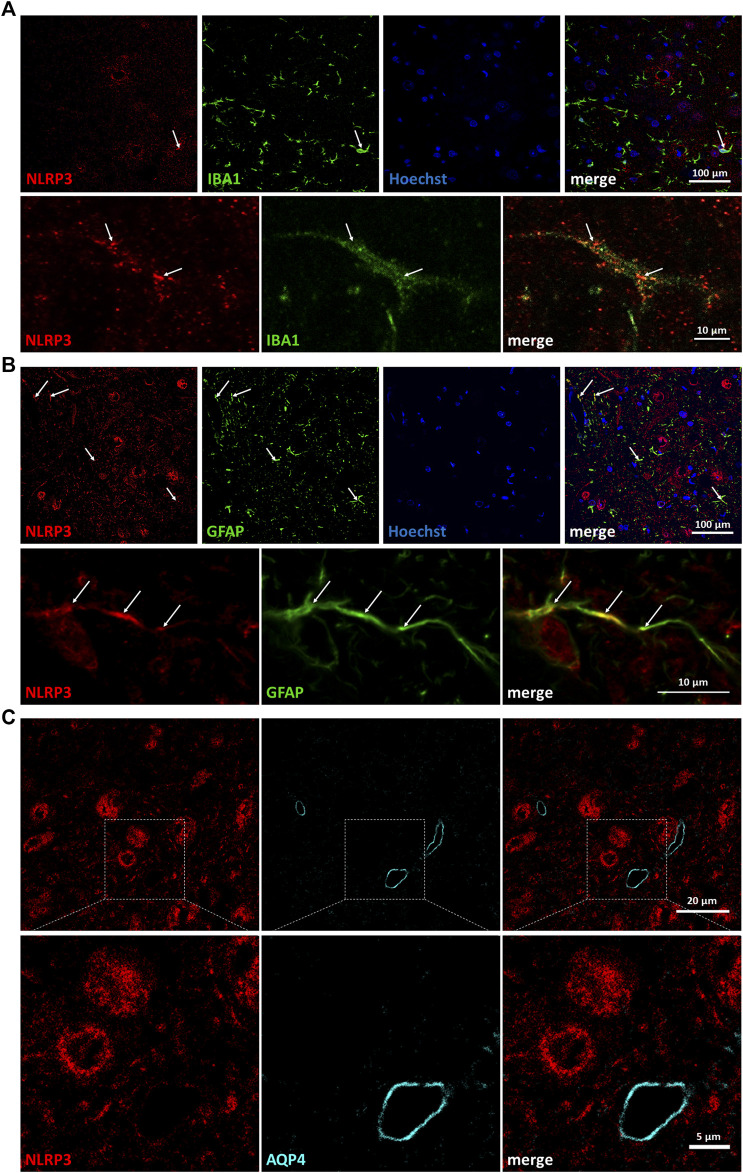
Partial co-localization of nucleotide-binding oligomerization domain-, LRR- and pyrin domain-containing protein 3 (NLRP3) with glial marker proteins but not with microvessels on the injured side. **(A)** Representative confocal super-resolution microscopy (STED) images from fluorescent immunostaining of NLRP3 and ionized calcium-binding adapter molecule 1 proteins on Balb/c mouse brain sections from the anatomical region of the hypoglossal nucleus after corresponding brain nerve axotomy. Nuclei were counterstained with Hoechst 33342. Top panels: lower magnification (scale: 100 µm); bottom panels: high magnification (scale: 10 µm). **(B)** Representative confocal STED images from fluorescent immunostaining of NLRP3 and glial fibrillary acidic protein proteins on Balb/c mouse brain sections from the anatomical region of the hypoglossal nucleus after similar brain nerve axotomy. Nuclei were counterstained with Hoechst 33342. Top panels: lower magnification (scale: 100 µm); bottom panels: high magnification (scale: 10 µm). **(C)** Representative confocal STED images from fluorescent immunostaining of NLRP3 and aquaporin-4 proteins on Balb/c mouse brain sections from the anatomical region of the hypoglossal nucleus after similar brain nerve axotomy. Top panels: lower magnification (scale: 20 µm); bottom panels: high magnification (scale: 5 µm). Arrows indicate co-localization of signals.

In order to identify further cell types that might respond with the upregulation of NLRP3 to peripheral nerve injury, we performed co-staining with ionized calcium-binding adapter molecule 1 (Figure 2A), a microglial marker, and glial fibrillary acidic protein (GFAP) (Figure 2B), an astroglial marker. Importantly, the majority of the microglia were not stained with the NLRP3 antibody, only a few microglial cells were NLRP3 positive. Similarly, NLRP3 was upregulated only in a small fraction of astrocytes, and astrocytic endfeet were largely excluded as demonstrated by the co-staining with the endfeet marker aquaporin-4 (AQP4) (Figure 2C).

### Subcellular Redistribution of Nucleotide-Binding Oligomerization Domain-, LRR- and Pyrin Domain-Containing Protein 3 and Co-localization With Inflammasome Component Apoptosis-Associated Speck-Like Protein Containing a Caspase Activation and Recruitment Domain

**FIGURE 3:**
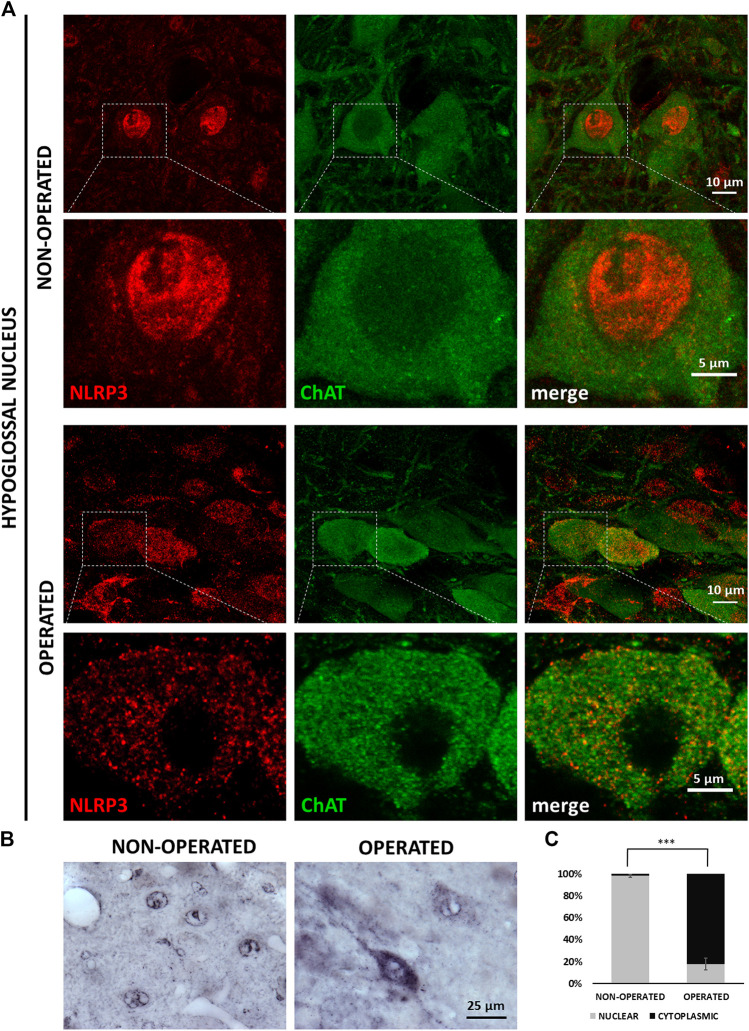
NLRP3 translocation from neuronal cell nuclei into the cytoplasm in response to XII. nerve axotomy. **(A)** Representative super-resolution microscopy [nucleotide-binding oligomerization domain-, LRR- and pyrin domain-containing protein 3 (NLRP3)] and confocal (ChAT) images on Balb/c mouse brain sections from the anatomical region of the hypoglossal nucleus from the non-operated and operated side after hypoglossal nerve axotomy. Top panels (on both the non-operated and operated sides): lower magnification (scale: 10 µm); bottom panels: high magnification (scale: 5 µm). **(B)** Representative immunohistochemistry staining images of NLRP3 protein on Balb/c mouse brain sections from the anatomical region of the hypoglossal nucleus, from the non-operated (left panel) and operated (right panel) sides after equivalent nerve axotomy (scale: 25 µm). **(C)** Quantification of intracellular distribution of NRLP3 staining in neurons from non-operated and operated sides of the hypoglossal nucleus. The graph shows average % ± SEM of neurons in which NLRP3 localized in the nucleus or the cytoplasm (n = 5 animals/group, *** = *p* < 0.0005).

Under control conditions, a significant part of NLRP3 staining could be seen in the nuclei of neurons. In response to the transection of the hypoglossal nerve, the staining appeared mainly in the cytoplasm, in parallel with weakening of the nuclear staining (Figure 3A). In order to visualize the changes more accurately and quantitatively, we performed IHC stainings which confirmed our observations obtained with fluorescence staining (Figure 3B). Quantitative analysis revealed that the ratio of neurons showing cytoplasmic NLRP3 was significantly higher on the injured side of the hypoglossal nucleus following axotomy (82.24 ± 5.38 vs. 1.95 ± 1.33%; injured side vs. contralateral side; *p* < 0.0005) (Figure 3C).

**FIGURE 4:**
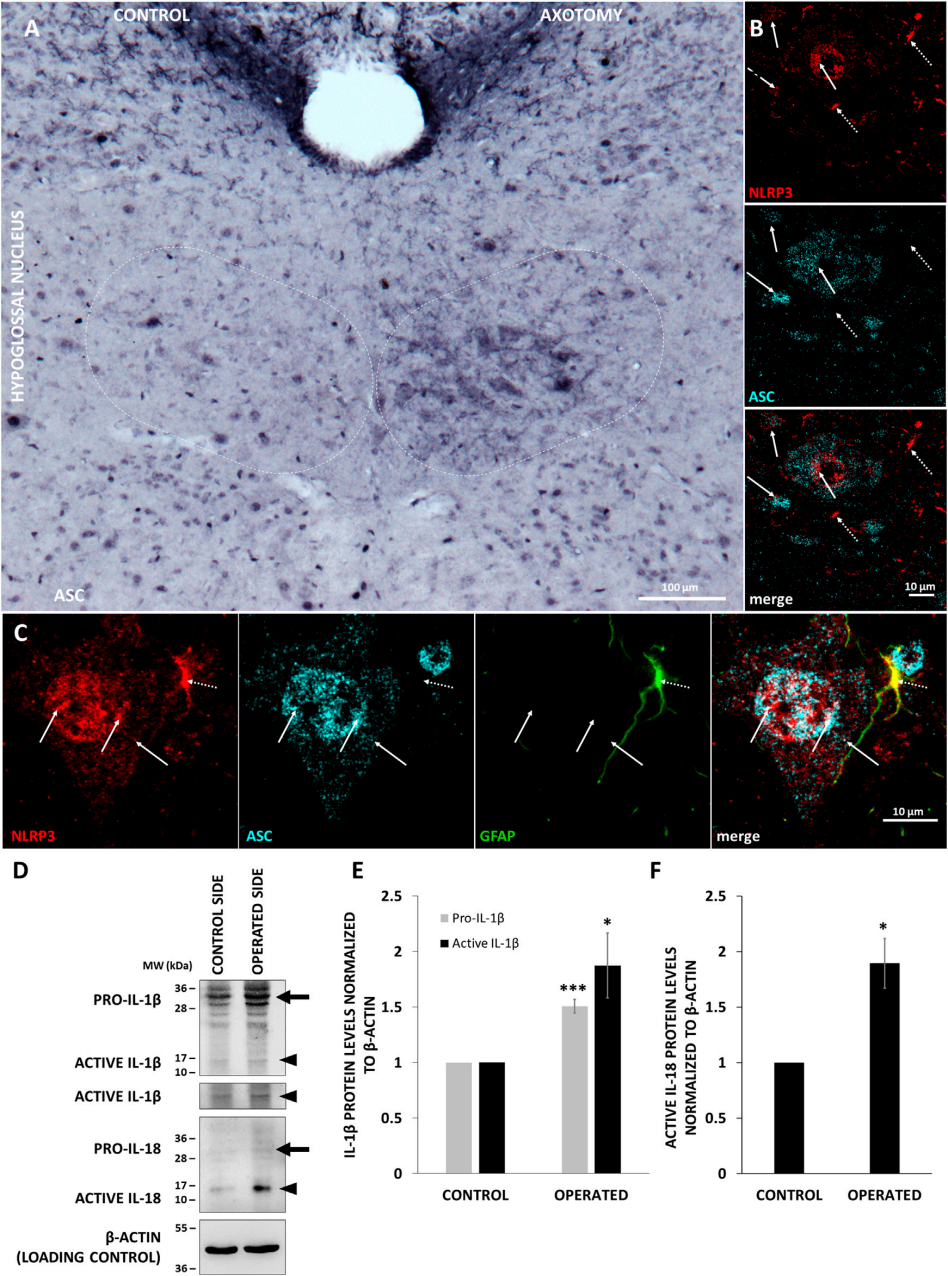
Co-localization of inflammasome components and production of active interleukin-1 beta (IL-1β) and IL-18 (IL-18) in the XII. nucleus. **A)** Representative immunohistochemistry staining images of apoptosis-associated speck-like protein containing a caspase activation and recruitment domain (ASC) protein on Balb/c mouse brain sections from the anatomical region of the hypoglossal nucleus after corresponding brain nerve axotomy (scale: 100 µm). **(B)** Representative confocal [nucleotide-binding oligomerization domain-, LRR- and pyrin domain-containing protein 3 (NLRP3)] and super-resolution microscopy (STED) (ASC) images on Balb/c mouse brain sections from the anatomical region of the hypoglossal nucleus after hypoglossal nerve axotomy (scale: 10 µm). Solid arrows indicate the co-localization of NLRP3 and ASC. Dashed arrows indicate NLRP3 without co-localization with ASC. **(C)** Representative confocal [NLRP3 and glial fibrillary acidic protein (GFAP)] and STED (ASC) images on Balb/c mouse brain sections from the anatomical region of the hypoglossal nucleus after corresponding brain nerve axotomy (scale: 10 µm). Solid arrows indicate co-localization of NLRP3 and ASC in the nucleus and cytoplasm of the cells. Dashed arrow indicates co-localization of NLRP3 with GFAP, but not with ASC. **(D)** Representative western blot images of IL-1β and IL-18 proteins in of the hypoglossal nuclei after unilateral axotomy of corresponding brain nerve. Arrows indicate pro-forms, arrowheads show active cytokines. **(E)** Quantification of pro- and active IL-1β expression based on the western blot analysis of the hypoglossal nuclei in n = 3 animals. The graph shows values normalized to *β*-actin levels and to control side (average ± SEM,* = *p* < 0.05; *** = *p* < 0.0005). **(F)** Quantification of active IL-18 expression based on the western blot analysis of the hypoglossal nuclei in n = 3 animals. The graph shows values normalized to *β*-actin levels and to control side (average % ± SEM, * = *p* < 0.05).

Activation of inflammasomes involves formation of a multiprotein complex, which includes binding of NLRP3 with the adaptor molecule ASC. Therefore, we performed co-localization analysis with the two proteins, and for a better resolution, we used STED microscopy. Similarly to NLRP3, ASC was also upregulated after nerve transection in the hypoglossal nucleus (Figure 4A). Staining performed with NLRP3 and ASC significantly overlapped mainly in neurons (Figure 4B). Interestingly, the co-localization could be observed in the nuclei as well (Figure 4C). Although NLRP3 could be detected in GFAP-positive cells as well, there was almost no co-localization with ASC (Figure 4C). We could not detect any co-localization of NLRP3 and ASC in microglial cells either (data not shown).

### Nucleotide-Binding Oligomerization Domain-, LRR- and Pyrin Domain-Containing Protein 3 Inflammasome Activation, Interleukin-1 Beta and Interleukin-18 Activation

To examine NLRP3 inflammasome activation, the protein levels of active IL-1β and IL-18, the main pro-inflammatory cytokines of the inflammasome pathway, were quantified with WB. These changes were evaluated in the hypoglossal nucleus following axotomy of the hypoglossal nerve, since more intense NLRP3 upregulation was observed here compared to the oculomotor nucleus. Axotomy resulted in a 1.507-fold increase of pro- IL-1β levels (*p* < 0.0005), indicative of the priming phenomenon. In addition, we observed a 1.873-fold increase of the active IL-1β protein levels in the operated hypoglossal nucleus compared to the control side (*p* < 0.05) (Figures 4D,E). Similarly, a 1.893-fold increase was observed in the active IL-18 levels after axotomy (*p* < 0.05) (Figures 4D,F).

### Inhibition of Nucleotide-Binding Oligomerization Domain-, LRR- And Pyrin Domain-Containing Protein 3 Upregulation by Diazoxide

**FIGURE 5:**
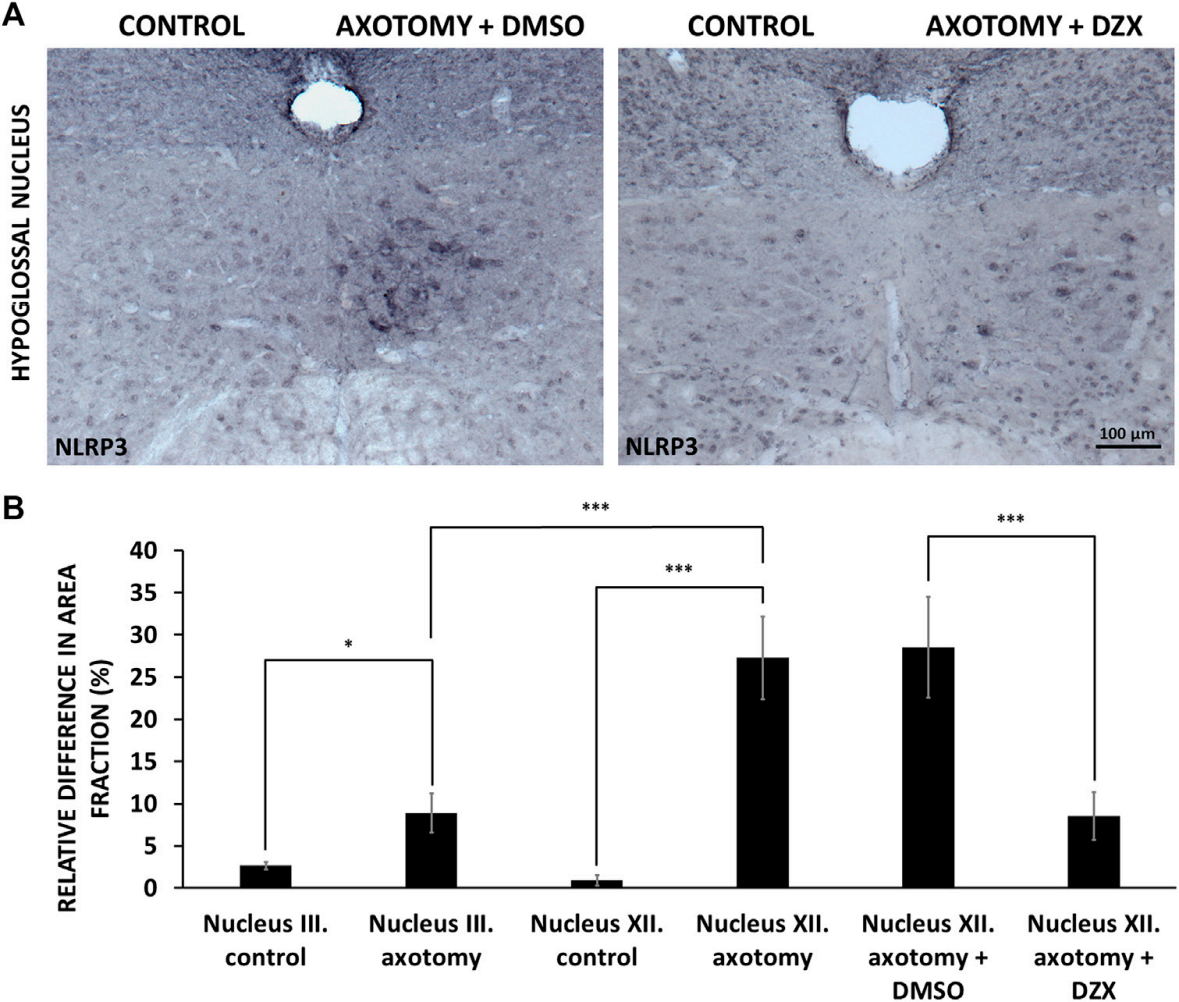
Effect of diazoxide on nucleotide-binding oligomerization domain-, LRR- and pyrin domain-containing protein 3 (NLRP3) protein expression in hypoglossal nucleus. **(A)** Representative immunohistochemical staining images of NLRP3 protein on Balb/c mouse brain sections from the anatomical region of the hypoglossal nucleus after axotomy and treatment with either vehicle [dimethyl sulfoxide (DMSO)] or diazoxide (DZX) each day for 4 days after surgery (scale: 100 µm). **(B)** Quantitative analysis of NLRP3 protein expression in the oculomotor (n = 4) and in the hypoglossal (n = 4) nuclei after corresponding nerve axotomy. Hypoglossal nerve axotomized mice were treated with either vehicle (DMSO; n = 3) or diazoxide (n = 4) each day for 4 days after surgery. Graph represents the relative difference in the staining area fraction (average % ± SEM) of the operated side compared to the non-operated side of the nuclei (* = *p* < 0.05; *** = *p* < 0.0005).

In order to investigate the role of mitochondrial injury in axotomy-induced NLRP3 upregulation, we treated experimental animals post-surgery for 4 days with diazoxide, an agent with proven neuroprotective effects, able to preserve mitochondrial function (Teshima et al., 2003). Diazoxide diminished the increase in NLRP3 expression observed in the non-treated group following axotomy in the hypoglossal nucleus, compared to the vehicle control (DMSO), as represented by NLRP3 staining (Figure 5A) and quantitative analysis (8.49 ± 2.84 vs. 28.49 ± 5.98%; injured + diazoxide vs. injured + vehicle; *p* < 0.0005) (Figure 5B). DMSO did not have any significant effect on axotomy-induced NLRP3 upregulation (Figure 5A,B).

## Discussion

Elaborate crosstalk between the nervous and the immune systems modulates a considerable number of pathological processes in the brain. Accumulating evidence suggests that interaction between damaged neurons and the immune system, especially the innate immune system, is pivotal in determining the outcome of neurological disorders (Trakhtenberg and Goldberg, 2011). Here we demonstrate that after acute lesion, motor neurons respond with upregulation of NLRP3, an important PRR and inflammasome component. NLRP3 upregulation and inflammasome activation have been largely acknowledged phenomena in CNS diseases (reviewed in Voet et al., 2019); however, microglia, the innate immune cells of the brain, have primarily been considered as resident cells involved in immunological processes of the CNS. Interestingly, in our model, motor neurons proved to initiate NLRP3 activation, while microglia were much less involved, despite their unequivocal role in the pathomechanism of neuronal injury-induced inflammation. Indeed, other NLRs not tested here, like NLRP1 or AIM2, may also become upregulated in microglia or neurons as well in response to axonal injury.

Although our finding that NLRP3 was highly upregulated in neurons was somehow unexpected, neuronal NLRP3 upregulation and inflammasome activation has already been detected in a few pathological conditions. For example, ischemic stroke was shown to activate both NLRP1 and NLRP3 inflammasomes in neurons through nuclear factor kappa-light-chain-enhancer of activated B cells (NF-κB)- and mitogen-activated protein kinase-dependent mechanisms (Fann et al., 2018). NLRP3 can be a mediator of inflammatory pain as well: chemical stimulation of the dura activated NLRP3 inflammasomes mainly in C-type neurons in the trigeminal ganglion (Chen et al., 2014). Furthermore, in the SOD1 (G93A) transgenic mice model of amyotrophic lateral sclerosis (ALS), NLRP3 activation was predominantly detected in degenerating neurons of the anterodorsal thalamic nucleus (Debye et al., 2016). Other studies also suggested activation of NLRP3 in neurons in injury and sterile inflammation (Chen et al., 2019; Voet et al., 2019). Nevertheless, in TBI, a mechanical injury model, NLRP3 upregulation was approximately similar in neurons, astrocytes and microglia (Liu et al., 2013), while in spinal cord contusion injury, NLRP3 was primarily found in microglia and neurons (Zendedel et al., 2015). However, to our best knowledge, inflammasome activation in the CNS after peripheral nerve injury has been unknown so far.

In our previous study, we have demonstrated that following acute nerve axotomy, microglia become activated to a different extent in the different motor nuclei of the brain, depending on the susceptibility of the respective nucleus to injury in chronic stress conditions (Nógrádi et al., 2020). For example, in the oculomotor nucleus, that is known to be resistant to degeneration in ALS, microglial activation was weaker than in the hypoglossal nucleus (Nógrádi et al., 2020), which is classically used as a model region of motoneuronal neurodegeneration in the CNS (reviewed in Nijssen et al., 2017). However, our current results indicate that microglial activation does not necessarily lead to inflammasome activation. This might have functional consequences, since specific microglia depletion had no influence on neuronal degeneration and axon regeneration in another acute nerve injury model (Hilla et al., 2017). On the other hand, the extent of microglial activation correlated with the level of motoneuronal NLRP3 upregulation in the two examined nuclei. Reduced NLRP3 upregulation after axotomy in the oculomotor nucleus compared to the hypoglossal nucleus might be coupled to the improved ionic buffering capacity of oculomotor neurons, especially to their ability to control calcium accumulation in mitochondria (Obál et al., 2006).

Under control conditions, we observed a strong NLRP3 staining in the cell nuclei. In response to nerve injury, NLRP3 was not only upregulated, but also translocated to the cytoplasm of motoneurons. Increased production of active IL-1β and IL-18 on the side of the injury suggests activation of inflammasomes, since cleavage of these cytokines is one of the best readout parameters of inflammasome activity (Broz and Dixit 2016; He et al., 2016; Zheng et al., 2020). Although the classical site of assembly of inflammasomes is the cytoplasm, a recent study revealed that six out of the 20 examined NLRs were detected in the nucleus as well. These include NLRP1, NLRP3, NLRP5, NLRP6, NLR family acidic transactivating domain (NLRA) and NLRC5 (Wang et al., 2016). Although we could clearly detect translocation of NLRP3 from the nucleus to the cytoplasm after nerve injury, a faint NLRP3 signal could still be detected in the nucleus, co-localized with ASC. Importantly, in certain cases, inflammasomes can be activated in the nucleus as well. Nuclear activation of interferon gamma-inducible protein 16 (IFI16) inflammasome could be detected during Kaposi sarcoma-associated herpesvirus infection in endothelial cells (Kerur et al., 2011). In the nucleus, activated caspase-1 may have other substrates than pro-IL-1β and pro-IL-18, e.g., sirtuin-1. Besides participating in the formation of inflammasomes, nuclear NLRs may have other functions as well, e.g., NLRP3 is a transcriptional regulator in T helper type 2 cells and a repressor of regulatory T cell differentiation (Bruchard et al., 2015; Park et al., 2020). It is not known yet, whether beyond serving as a receptor in inflammasomes, NLRP3 has any regulatory functions in neurons.

Several therapeutic approaches have proved that inhibition of the NLRP3 pathway could successfully prevent or slow down neuronal loss in neurodegenerative disorders (Zhou et al., 2016; Dempsey et al., 2017; Gordon et al., 2018). Based on this, reduction of NLRP3 activation seems to be a promising point of intervention in acute injury conditions as well. Diazoxide, an activator of the mitochondrial ATP-dependent K^+^ channel, which readily crosses the blood-brain barrier, is known to reduce mitochondrial dysfunction (Domoki et al., 2004) and to protect neurons during ischemia/reperfusion injury (Lei et al., 2018). Opening of mitochondrial K^+^_ATP_ channels results also in activation of anti-apoptotic mechanisms in neurons (Liu et al., 2002).

Diazoxide could also block microglial activation and reduce mitochondrial swelling in neurons following nerve axotomy in our previous experiments (Nógrádi et al., 2020). Interestingly, such effect on microglial mitochondrial morphology was not observed. Furthermore, diazoxide could reduce NLRP3 activation during cerebral ischemia/reperfusion injury through protecting mitochondria (Zhe et al., 2018; Gong et al., 2018). Indeed, mitochondrial dysfunction has a pivotal role in initiation and activation of the NLRP3 inflammasome (Liu et al., 2018), thus the ability of diazoxide to reduce neuronal mitochondrial injury could be a major pathway in dampening NLRP3 upregulation following acute nerve injury.

In our acute nerve injury model, diazoxide was able to block NLRP3 increase in a post-surgery treatment paradigm. As the neuroprotective effect of diazoxide has already been established (Lei et al., 2018), our results raise the point whether blockade of NLRP3 increase might act as a contributing factor to the neuroprotective properties of diazoxide in acute nerve injury. Also, as diazoxide could dampen the axotomy-induced microgliosis, it is possible that this effect, at least in part, could be mediated through the reduction of mitochondrial injury and NLRP3 activation. Therefore, our observation further strengthens the significance of the anti-inflammatory properties of diazoxide from a therapeutic point of view.

## Data Availability Statement

The original contributions presented in the study are included in the article/supplementary material, further inquiries can be directed to the corresponding author.

## Ethics Statement

The animal study was reviewed and approved by Regional Animal Health and Food Control Station of Csongrád County.

## Author Contributions

BN, ÁN-T, and IAK designed research study; BN, ÁN-T, MK, KM, and RP performed research; RP, LS, IW, and IAK analyzed the data; LS and IAK supervised research; BN, ÁN-T, IW, and IAK drafted the manuscript; all authors approved final version.

## Funding

This work was partially supported by the NKFIH (National Research, Development and Innovation Office) through the GINOP-2.3.2-15-2016-00001, GINOP-2.3.2-15-2016-00034, GINOP-2.3.2-15-2016-00020, FK-124114, K-135425, and K-135475 programs; the ÚNKP-19-2-SZTE-92, ÚNKP-20-2-SZTE-68, and ÚNKP-20-4-SZTE-138 New National Excellence Program of the Ministry for Innovation and Technology of Hungary; the NTP-NFTÖ-20-B-0192 National Talent Program of the Hungarian Ministry of Human Capacities and the Szeged Scientists Academy under the sponsorship of the Hungarian Ministry of Human Capacities (EMMI:11136-2/2019/FIRFIN). Support of UEFISCDI (Executive Agency for Higher Education, Research, Development and Innovation Funding; project code: PN-III-P1-1.1-TE-2019-1302) is also acknowledged.

## Conflict of Interest

The authors declare that the research was conducted in the absence of any commercial or financial relationships that could be construed as a potential conflict of interest.
